# Measuring user engagement with low credibility media sources in a controversial online debate

**DOI:** 10.1140/epjds/s13688-022-00342-w

**Published:** 2022-05-16

**Authors:** Salvatore Vilella, Alfonso Semeraro, Daniela Paolotti, Giancarlo Ruffo

**Affiliations:** 1 Department of Computer Science, University of Turin, Turin, Italy; 2 ISI Foundation, Turin, Italy

**Keywords:** Misinformation, Disinformation, Information diffusion, Immigration, Online social networks

## Abstract

We quantify social media user engagement with low-credibility online news media sources using a simple and intuitive methodology, that we showcase with an empirical case study of the Twitter debate on immigration in Italy. By assigning the Twitter users an *Untrustworthiness* (*U*) score based on how frequently they engage with unreliable media outlets and cross-checking it with a qualitative political annotation of the communities, we show that such information consumption is not equally distributed across the Twitter users. Indeed, we identify clusters characterised by a very high presence of accounts that frequently share content from less reliable news sources. The users with high *U* are more keen to interact with bot-like accounts that tend to inject more unreliable content into the network and to retweet that content. Thus, our methodology applied to this real-world network provides evidence, in an easy and straightforward way, that there is strong interplay between accounts that display higher bot-like activity and users more focused on news from unreliable sources and that this influences the diffusion of this information across the network.

## Introduction

The era of digital media that unfolded during the second half of the 20th century has forced rapid and drastic changes upon the news media landscape. For several decades more traditional media have been challenged by the rise of digital-born media that have gained audience and attention, especially through blogs and social media platforms [1]. This proliferation of new actors allows for the coexistence of a vast plurality and diversity of voices, a media pluralism generally considered crucial for the well being of a democratic state. Access to different opinions and ideas is often referred as one of the basic rights of citizens to freely form their own informed opinions.^1^ On the other hand, a distrust for traditional forms of information sources has grown over the years and it has been amplified during the latest crisis caused by the COVID-19 pandemic.^2^

These dramatic and global changes in the news media ecosystem have increased the complexity of both the consumption patterns and the reliability assessment of news. A report promoted by the Council of Europe says that “information pollution at a global scale; a complex web of motivations for creating, disseminating and consuming these ‘polluted’ messages; a myriad of content types and techniques for amplifying content; innumerable platforms hosting and reproducing this content; and breakneck speeds of communication between trusted peers” create a global information problem that is difficult to quantify but can be addressed by tackling a number of issues. These include the implications of communication bubbles and the fact that different groups, especially on social media, fail to share a sense of reality based on facts [2]. Even if “fake news” related phenomena, such as disinformation, misinformation, propaganda, unverified rumours, poor reporting, and hateful and divisive messages, are nothing new, these issues have been recently taken into serious consideration at both scientific and political levels. Many national and supranational institutions are looking into the related technical and ethical problems because all kinds of mis- and dis-information—genuine or fabricated, malicious or benign—can ultimately influence political agendas. This has serious implications for public discussions of wide ranging topics, from public health (such as the pressing issue of the COVID-19 infodemic) to climate change [1], from economics to immigration [3].

We seek to evaluate the engagement of social media users with different sources of news, particularly with *unreliable* media outlets, meaning those that are recognised by multiple watchers as consistent publishers of fabricated or inaccurate news or possible followers of political agendas. Toward this aim, we define a simple measure of online user engagement with *reliable* and *unreliable* news media based on the frequency with which a piece of news is re-shared in a digital social network. We then evaluate this measure in the context of an empirical case study and validate it with other markers of information pollution, such as the presence of *social bots* or the (in)ability of news to diffuse over many different groups of users, to gain insights into a specific controversial topic, namely the public debate on Twitter around migrants and politics in Italy.

### Related work

Much scientific effort has gone into the detection and classification of different kinds of disinformation promoted by digital news articles. The approach generally involves the application of machine learning techniques to perform supervised classification of text, exploiting training sets labelled by humans on the dichotomy *real-fake* news [4–6] and on more diverse target variables that deal with, for example, the partisanship and writing style of textual excerpts [7] or the emotional analysis of political statements and claims that were previously labelled as true or false by fact-checkers [8], and many more. While the methods can depend on factors such as the performances of the chosen architecture or the quality of the training set, they allow researchers to focus on the individual pieces of media content (e.g., news articles or social media posts) to assess their truthfulness.

It is also possible to analyse the context at a higher level, focusing on media outlets rather than on individual news articles. One of the most common ways of selecting sources is by hand-picking a set of news media outlets that are well-known disinformation spreaders. The selection is usually done by referring to one or more of the many existing services that monitor the quality of information and debunk viral fake news. This or similar approaches are followed by many [9–12], including us as detailed in Sect. 2.1. Lazer et al. [13] state that they “*advocate focusing on the original sources—the publishers—rather than individual stories, because we view the defining element of fake news to be the intent and processes of the publisher*.” Notably, there is no trivial one-to-one correspondence between fake-news sources and digital media outlets, and legacy outlets are not exempt from publishing inaccurate news.

However, focusing on sources rather than on individual stories comes with advantages. Particularly, it allows expansion from the very specific phenomenon of fake news to more complex and multi-faceted issues, that include many different aspects such as fake-news, propaganda, lies, conspiracies, rumours, hoaxes, hyper-partisan content, falsehoods, and manipulated media. There are also drawbacks to consider: The complexity and diversity of these phenomena make them particularly challenging to synthesise and to unify under a single label. Discriminating between media outlets is not a trivial task and watch-lists are not easy to maintain or even to compile in the first place. Fact checkers need to take into account subtle aspects such as the context, the maliciousness of the publisher, and the temporal consistency of the act, which makes it an extremely delicate job.

After introducing a simple methodology to measure user engagement with low-credibility media content on an online social network, we test it on an empirical case study. Specifically, we apply our methodology to an analysis of Twitter user engagement with reliable and unreliable media content within the specific context of the Italian online debate over immigration. The online debate around migration has been studied recently in several national and cross-national contexts. Some scholars have observed that the topic of migration is often extremely polarising [14, 15], fragmented between shades of slightly different opinions [3, 16], and a display of very high level “mediatization” [17], with influential politicians and media outlets involved in the discussions [18]. Disinformation in such discussions can be instrumental in targeting both politicians [19] and migrants [19, 20] and the general attitude can depend on the particular country or events under examination [21]. In general, as this is a strongly politicised topic, it is not exempt from effects similar to those that disinformation has had on other similar topics. The prevalence of disinformation content in different online debates has been studied [9, 10, 22, 23]. In [22] the authors study a context very similar to the one we study here, adopt a comparable approach in the selection of content, and find evidence of connections between the Italian disinformation sources and other European counterparts. They find the majority of such content is being spread in the Italian conservative and far-right political environment.

Another important aspect of studying the spread of malicious content online is the presence of bot-like activity. A *social bot* can be defined as “*a computer algorithm that automatically produces content and interacts with humans on social media, trying to emulate and possibly alter their behaviour*” [24]. The impact of social bots has been assessed in many different contexts, following different approaches. Results are not always perfectly aligned as they strongly depend on their particular study cases. There is, though, general agreement on the fact that they influence the conversation to varying extents and, particularly, that there is strong interplay between bots and humans that is crucial in the virality of content [25–27]. Bots can often contribute negatively to the discussion [28], disrupting communications [29] or increasing polarisation [27]. Still, their contribution is not always as evident. In other experimental settings, even though a clear presence of bots had been found, the impact on the conversation appeared limited [30, 31]. Vosoughi et al. [25] make a crucial contribution to understanding that the role of bots is often non-trivial and deeply rooted into their interaction with human users. Finally, Shao et al. in [26] have produced a considerable amount of empirical evidence aiming at better understanding the role of social bots in the spread of low-credibility content. They found that social bots play a disproportionate role in spreading articles from low-credibility sources, amplifying the diffusion of such content in early spreading, before an article goes viral. Most importantly, bots target users with many followers through replies and mentions, exploiting human vulnerabilities to this kind of manipulation: Real users are fooled as they are likely to re-share content posted by bots. Hence, bots play a fundamental role in diffusing disinformation and malinformation, even though influential users that are targeted and easily manipulated into re-sharing low-credibility posts should probably be blamed the most.

Finally, the connection between political diversity among a specific website’s users and the quality of the news presented by the website has been studied recently by Bhadani et al. [32]. Authors use news source reliability ratings from domain experts and web browsing data from a sample of US residents and show that websites with less politically diverse audiences have lower journalistic standards. These results can be exploited in designing better algorithmic ranking decisions to improve the quality standards of news proposed to users. This connection between quality and diversity is quantifiable, to some extent, and content can be diffused over many different politically characterised communities, as we explore in this paper.

### Research questions and our contribution

A quantitative measure of how much users of online social networks engage with news articles with questionable reputations and how much this engagement is connected to the presence of bot-like behaviour would provide additional insights on the ecosystem of online social media, as well as on the consumption and diffusion of media content in polarised debates. We aim with our methodology to answer these related research questions:

- *R1*: How frequently is content with different levels of credibility shared on online social media? Is it possible to identify user patterns of news consumption that concur, at a coarse-grained level, with community engagement with unreliable media content?
- *R2*: Is there a statistically significant portion of bot-like activity within these news consumption patterns?
- *R3*: Do the above features influence diffusion of content over many different communities on a network?
- *R4*: How does the probability of *success* (in terms of spread) of a piece of content change in light of these features?

To answer these questions we introduce an “Untrustworthiness” index, a measure of how much a single user engages with content from low credibility media sources. We then apply this measure in to a specific case study to characterise a large network of social media users, combining our metric with a third-party tool to quantify the presence of social bots or accounts showing bot-like behaviour. Finally, we track the diffusion of news articles on the case-study network.

## Methods

Users sharing content on an online social media platform are referred to here as *nodes* in an interaction network. For these nodes, we assume we have the online digital identity (e.g., a Twitter user handle) and can track information shared by each one (e.g., original posts, re-tweets, etc.).

### Untrustworthiness index

To answer research question R1, we define the *Untrustworthiness* index and use it to measure how much each user who created or retweeted at least one post containing a URL linking to an external resource engages with unreliable media outlets. This approach relies on external annotation of the media outlet credibility level and, most importantly, does not focus on the veracity of individual pieces of news, but rather on the reputation of the publisher.

Let $L^{\oplus}$ and $L^{\ominus}$ be, respectively, reliable and unreliable lists of externally-annotated media-outlet web domains. Typically, *unreliable* news outlets are those flagged as consistent spreaders of disinformation by independent annotators. News published by such outlets can be shared by users on social media in the form of URLs pointing to the relevant web domains. Let *V* be the set of users and $T_{v}$ the total number of posts produced by user $v \in V$ that contain a URL from either of the two lists. If we consider $T^{\ominus}_{v}$ and $T^{\oplus}_{v}$ to be the number of posts produced by user *v* that contain a URL from $L^{\ominus}$ and $L^{\oplus}$, respectively, then

$$ T_{v} = T^{\ominus}_{v} + T^{\oplus}_{v}$$

will hold. We can easily calculate the ratio

$$ R_{v} = T^{\ominus}_{v}/T_{v}$$

of posts produced by *v* that contain a URL of an unreliable media source over the total number of posts that contain any URL (from both reliable and unreliable sources). To assess an account’s reliability, not only in terms of this ratio, but also as a function of its activity, we define the *Untrustworthiness* of user *v* as the harmonic mean of $R_{v}$ and $T_{v}/max(T_{v})$:

1 $$ U_{v}= \biggl(\frac{\frac{T}{T_{v}}+\frac{1}{R_{v}}}{2} \biggr)^{-1}, $$

where $T= \max_{v \in V}(T_{v})$ is a normalisation factor that takes into account the maximum volume of activity in the dataset, that is, the highest number of tweets that contain a URL by an individual user. This way, we avoid over weighting accounts that appear sporadically or that have very little activity relative to the rest of the dataset.

*U* is not merely a count of shares of posts that are considered unreliable. It provides a simple yet quantitative way to assess the level of engagement of each user with media outlets with different levels of credibility. Users with higher *U* that tweeted hundreds of times are likely to be consistent spreaders of less accurate information (including disinformation); users with lower *U* that tweeted reliable news in a consistent fashion are likely to be only occasional sharers of low-quality information.

### BotScore

To study the contribution of bot-like users to the diffusion and spread of news, we use existing tools to evaluate their pervasiveness. In general, identifying online social media bots remains a difficult task but the *Botometer* service [33] is a valuable, constantly upgraded and validated tool that we can take advantage of. The Botometer is developed by the researchers at the Observatory on Social Media (OSoMe) at Indiana University. It receives a Twitter user id as input and returns a set of scores that assess the “botness” of the corresponding account, leveraging a set of different classifiers trained to identify several types of bots, such as spammers, astroturfs, and financial bots. The Botometer models have been trained using different feature sets that include network metrics and text based attributes. The Botometer uses a *language-independent* classifier to provide an *overall raw score* (henceforth called “BotScore”) in the interval $[0,1]$ that indicates the likelihood that an account is controlled by a bot (see Fig. 2(c) for two illustrative examples).

### Diffusion of news-related content in an online social network

Users in a online social network are known to share content from different kinds of media outlets. If the network of interactions is known, it is possible to track of the diffusion of every URL shared on the network. In a social media network, such as a re-tweet network on Twitter, every URL that is shared on the network has one or more original posters (OPs). OPs are the users that inject a given piece of news into the network through a tweet for the first time. Other users can then *retweet* the original and initiate diffusion of the URL on the network. Online social networks, especially Twitter, are prone to display topological features, such as groups or communities, that reflect the different opinions and levels of homophily among the various nodes. If we assume that our network presents different communities in the context of a specific topic, we can then study URL diffusion chains to understand the sharing patterns across the different communities. Let $\text{URLs} = \{\text{url}_{1}, \text{url}_{2}, \ldots , \text{url}_{m} \}$ be the set of all the URLs that have been shared on a network. Then, we can quantify how *heterogeneous* the reach of a URL is, in terms of how many different communities it reaches, by defining an entropy measure [3],

2 $$ H({\text{url}_{i}})= -\sum_{c \in C} s_{c}(\text{url}_{i}) \ln \bigl(s_{c}( \text{url}_{i})\bigr) , $$

where $s_{c}(\text{url}_{i})$ is the number of shares of each $\text{url}_{i} \in \text{URLs}$ in each community *c*. This enables us to assign each ${\text{url}_{i}} \in \text{URLs}$ a quantity that provides a measure of how much the external content is spread across different clusters or, instead, how much it remains trapped in a “bubble.”

By combining the methods proposed in Sects. 2.1, 2.2, and 2.3 we aim to answer the research questions highlighted in Sect. 1.2. We provide a quantitative characterisation of engagement with unreliable content and a description of the interplay between accounts with different *U* and BotScores in the diffusion of media content.

## Case study: the Italian public debate on Twitter around the immigration issue

In this section we describe the application of our methodology to a specific case study that is particularly relevant because it is shaped by many complex aspects, from politics to communication patterns induced by social media design.

### Dataset and retweet network

We apply our methods to the Tweets in Italian on Immigration (TWITIMM) dataset [3]. TWITIMM includes about 6 millions tweets in Italian, published by a set *V* of more than 200,000 unique users, and spans from August 2018 to August 2019. This is the year of the so-called “first Conte’s Government,” when Prime Minister Giuseppe Conte was leading a right-wing majority that put the fight against illegal immigration at the top of the government agenda.

From TWITIMM it is therefore possible to build a retweet network $G=\{V,E\}$ whose nodes $s,t \in V$ are the Twitter users, and directed link $l = (s,t) \in E$ is established when *s* has retweeted a tweet created by *t* at least once. Every link *l* has a weight *w* that represents how many times user *s* has retweeted content created by user *t*. The resulting network contains more than 200,000 users and 2 millions edges. A comprehensive analysis of this network has been conducted in [3], where one of the main results is from the study of the community structure that reflected the divisions of the Italian political landscape with respect to immigration at that time. “Common” users cluster around the accounts of well-known politicians, and like-minded journalists and media outlets. For the benefit of the reader, the community structure studied in [3] is shown in Table 1.

**Table 1:** The communities found in *G* in terms of size, internal link density, political leaning and/or general characterisation, and inferred stance toward immigration [3]

Community ID	Size	Internal link density	Political area / characterisation	Inferred stance toward immigration
RT1	116,831	1.5⋅10^−3^	Left, Centre-left, Democrats	Positive
RT2	34,174	1.93⋅10^−2^	Right, Far-right, Hoaxers, News Media	Negative
RT3	27,845	2.4⋅10^−3^	*League* party, Right, News Media	Negative
RT4	9553	3.5⋅10^−3^	5 Stars Movement, News Media	Mixed
RT5	9225	2.4⋅10^−2^	News Media, All News outlets	Neutral

We also refer to the TWITA dataset [34].^3^ It is a collection of Italian tweets without any topic filtering, so we use it as a neutral baseline to test the robustness of the results of the application of the Untrustworthiness index.

### Application of the untrustworthiness index

**Figure 1:**
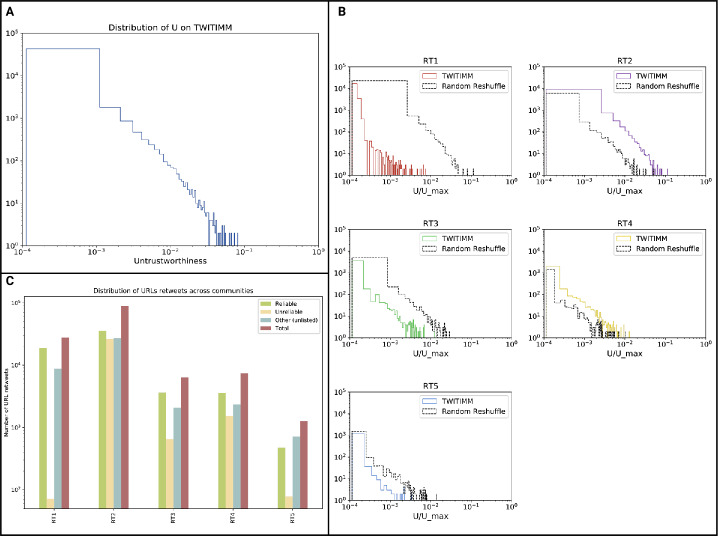
(**A**) Distribution of the Untrustworthiness index of Twitter accounts in TWITIMM, i.e., accounts involved in the immigration debate. The numbers, weighted by the activity of the users, tend to be very low; in logarithmic scale we can see that the vast majority of the users have low U, but the right tail of the distribution decreases slowly, highlighting a core of high-*U* users at the very end of the distribution; (**B**) Distribution of *U* disaggregated by clusters and tested against a randomly reshuffled community partitioning. All the distributions are statistically non-random according to the Mann-Whitney test ($p \leq 10^{-4}$). We see how RT2, identified as an anti-immigration cluster, clearly exceeds the average number of high-*U* users by far, showing a higher and longer right tail than the other communities, suggesting that the prevalence of low credibility media outlets in this cluster is much higher. (**C**) Shares of URLs disaggregated by community and by type of URL. Links to *reliable* and *unreliable* outlets are those that concur with the calculation of the Untrustworthiness score (i.e., users showing a striking preference for one type or the other can be classified, respectively, as trustworthy and untrustworthy), while the category *other* includes all URLs that do not belong to our lists, thus not contributing to the calculation of U. We observe very different sharing patterns between the communities, with users in some sharing a significantly higher number of reliable URLs, while others show smaller differences between reliable and unreliable media outlets. Differences have been statistically tested as shown in Tab. 2

To apply the methodology described in Sect. 2.1 to the *G* network, we first define the two lists $L^{\oplus}$ and $L^{\ominus}$ to determine the reliable and unreliable media outlets. For $L^{\ominus}$, we can rely upon the blacklisted set of web sites available from two main debunking sites in Italy, “*butac.it*” and “*bufale.net*.” From them we obtained a selection $L^{\ominus}$ of 25 websites that were consistent in publishing political mis- and dis-information and still active as of August 2019. We then used the Audiweb 2019 reports^4^ to select the top 100 information outlets by digital accesses, filtered out blacklisted sites already in $L^{\ominus}$, and obtained $L^{\oplus}$.^5^ The two complete lists can be seen in App. A.1, where we also provide some additional details about the frequency of the URLs as a function of their popularity ranking and the 15 most re-shared web domains. In Fig. 1(c) we show the distributions of URLs pointing to different classes of outlets.

Once we defined the lists, we tracked every URL shared by the users in TWITIMM and assigned a *U* value to every user that shared news from media outlets in either $L^{\oplus}$ or $L^{\ominus}$. If a URL could not be classified as $L^{\oplus}$ or as $L^{\ominus}$, as defined above, we labeled it “Other”and kept it out of the Untrustworthiness Index calculation.

Figure 1 shows an overview of the results of the application of the Untrustworthiness index on the TWITIMM dataset. Particularly, in Fig. 1(a) we see the general distribution of *U* across the various users. The vast majority of them lie on the left side of the distribution, but the tail decreases slowly, highlighting that there are a number of high-*U* users at the very end of the distribution.

We tested the robustness of *U* on the TWITA dataset to check the hypothesis that the score calculated on our dataset, *G*, built on immigration-related words, could overestimate or underestimate the presence of low-credibility sources among the posts. We computed the *U* score over the same one-year period, from August 2018 to August 2019, on a subset of users found in both datasets (TWITIMM and TWITA). The results are consistent with those found in our dataset: 98.7% of the nodes have an overall *U* in TWITA which is within a ±0.01 range of their own *U* in TWITIMM.

In Fig. 1(b) we disaggregate the distribution of *U* into the communities found in *G* (described in Table 1) and test the disaggregated distributions against a random reshuffling. The distribution of *U* in cluster RT2 is strikingly different from the others. *U* scores in RT2 are much higher, with a relevant number of users with high Untrustworthiness. RT2 is also the second largest community (see Table 1) and identified as an anti-immigration cluster [3]. Its higher degree nodes correspond to accounts controlled by politicians, newspapers, and celebrities who are publicly and vocally against immigrants and often in close liaison with nationalist and right-wing parties. On the contrary, RT1 has very few users in the right tail of the distribution (with high *U*) even though it is by far the largest community. Mainly, the distributions seem to show a characteristic “untrustworthiness fingerprint” for each of the clusters, and this hypothesis holds against a randomisation of the community assignments (Fig. 1(b)). The differences between the distributions for each community and their random counterparts are all statistically significant (Mann-Whitney test, $p \leq 10^{-4}$). RT2’s original data also shows that $BotScore$ scores are constantly higher than the expectation from a random reshuffle, while RT1 shows the opposite behaviour.

Finally, in Fig. 1(c) we characterise the five communities by the number of retweets for every kind of URL found in the dataset, $L^{\oplus}$, $L^{\ominus}$, or neither. As expected, RT2 stands out due to the proportion of retweets towards unreliable media outlets compared to the total, a ratio that is generally lower for the other communities and particularly for RT1. These differences have been statistically tested, as shown in Table 2.

**Table 2:** P-values of community-pairwise Mann-Whitney tests for the data shown in Fig. 1(c). Wherever $p<0.05$ we can reject the null hypothesis of the test and state that the distributions of URLs between the two communities are statistically different

	RT1	RT2	RT3	RT4	RT5
RT1	-	0.02	0.44	0.06	0.33
RT2	0.02	-	0.02	0.03	0.02
RT3	0.44	0.02	-	0.03	0.33
RT4	0.06	0.03	0.03	-	0.02
RT5	0.33	0.02	0.33	0.02	-

### Application of the BotScore

**Figure 2:**
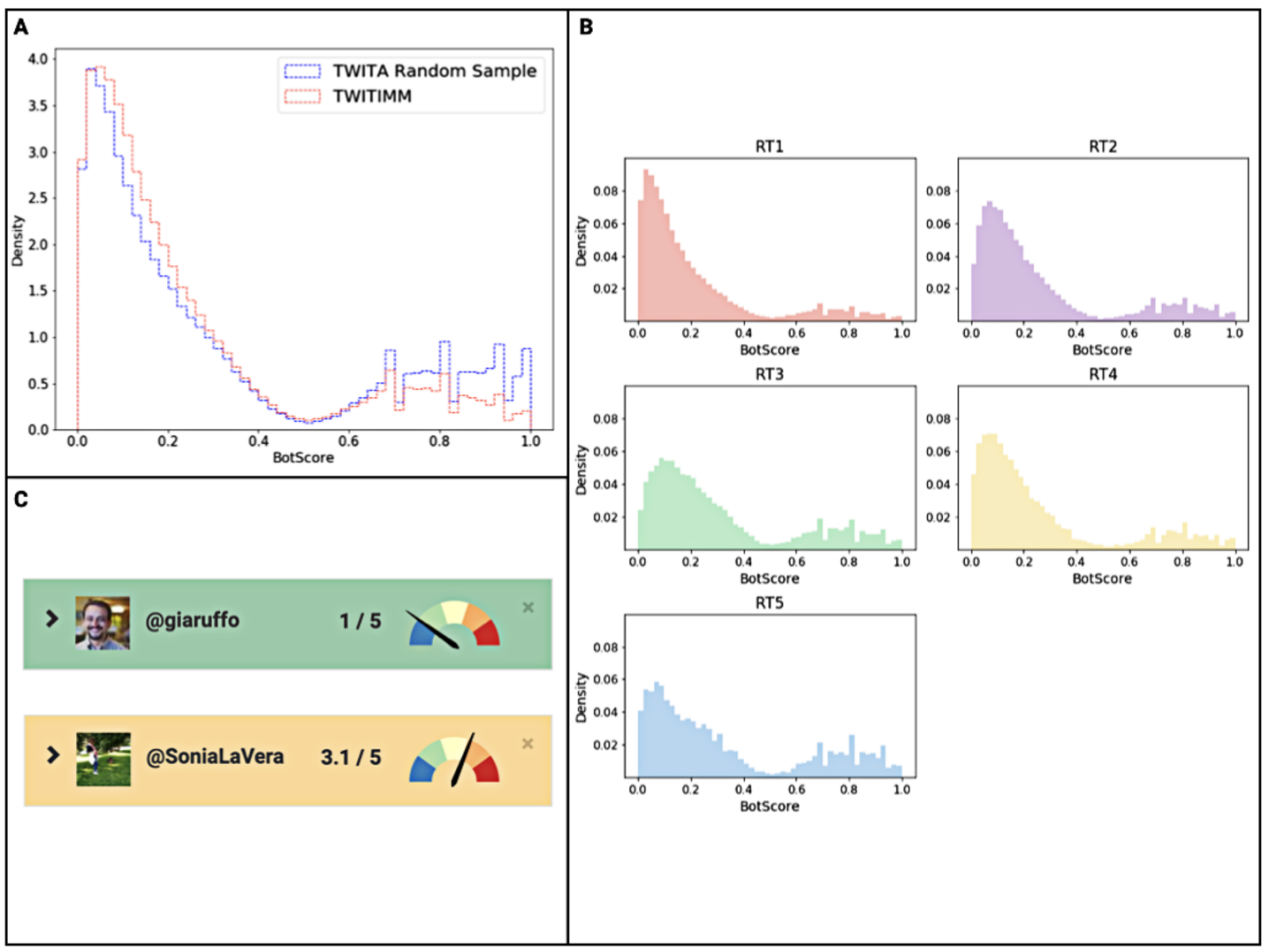
(**A**) Distributions of BotScores of Twitter accounts in TWITIMM, i.e., accounts involved in the immigration debate, compared to a random sample of Italian tweets produced during the same period, and equivalent in size; (**B**) BotScore distribution of all the users in the network, disaggregated by community. We can see that the right tail of the distribution—accounts showing high BotScores—is generally much lower than the left tail. This is particularly true for RT1; (**C**) Example of BotScore calculated on two different Twitter accounts: one of the authors of the paper (top) and the account with highest *U* of the TWITIMM dataset (bottom). Results from https://botometer.osome.iu.edu using non-raw scores that span from 0 to 5

Research question R2 is answered by using the Botometer service on our dataset, as described in Sect. 2.2. In Fig. 2(a) we plot the distribution of the BotScore obtained by running the Botometer on the users in *V*. In the same figure, we also show a baseline distribution from a random sample of accounts of size equal to $|V|$. We extracted this sample of Italian Twitter users from the TWITA database and kept the same temporal distribution of daily unique users found in TWITIMM. The two distributions are significantly different according to the Mann-Whitney test ($p \leq 10^{-4}$). Quite interestingly, the predominance of accounts likely controlled by humans over accounts that show some degree of automation is more pronounced among the tweets related to the immigration debate than among the randomly selected tweets related to different topics. Although we do not have an explanation for this, we suspect divisive topics are more engaging for real users than are other conversations.

It is important to stress that this is not a task of binary classification. We are not discriminating between bots and non-bots, and we are not arguing that an account whose BotScore is above an arbitrary threshold is guaranteed to be a bot. As noted by Cresci et al. [35] in 2017, neither Twitter, nor humans, nor up-to-date tools were capable of accurately detecting a novel family of social spam-bots. This observation is still valid today, and bot identification is destined to remain a moving target for many years to come. However, our purpose is to draw general statistics over the distribution of the BotScore in our dataset as markers of possible bot-like activity. To assess the Botometer’s performance, we followed the guidelines suggested in [36] where the authors point out a series of steps that should be taken to mitigate some drawbacks that characterise many automated tools for bot detection. In particular, we carried out a manual annotation on a stratified sample of accounts from our dataset to validate the scores obtained through the Botometer. The results are encouraging because more than half of the alleged bots according to the human annotation are above $\mbox{BotScore} > 0.36$, a symbolic threshold that, in our dataset, accounts for 80% of the users. Furthermore, we checked the temporal consistency of the Botometer’s annotation and found a strong linear correlation (Pearson’s $\mathrm{coefficient} = 0.84$, $p=2.8\cdot 10^{-38}$) between the annotation on the same set of accounts in December 2020 and September 2021. Further details on this check can be found in Appendix Sect. A.2.

Finally, as we did for the Untrustworthiness Index, we plotted the BotScore distributions disaggregated by community (Fig. 2(b)). The BotScore distribution are characterised differently with respect to *U* scores. We were not able to identify one or two communities that stand out among the others in terms of a much higher presence of bots. Nonetheless, we can say that there are clear differences among the clusters. As for Untrustworthiness, the distributions are statistically significantly different from a random baseline (Mann-Whitney test, $p \leq 10^{-4}$). In all the communities, we observe evidence that accounts do not show high bot-like activity (the left side of the distribution is, in general, much higher than the right side).

### Diffusion of URLs and impact of the original posters

**Figure 3:**
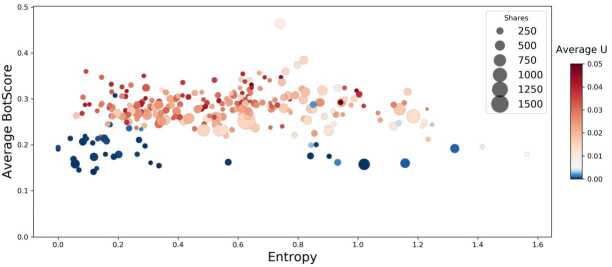
BotScore as a function of the entropy: each dot is a URL. BotScore and Untrustworthiness by URL are computed as the average values of the scores of the users retweeting each URL. Also, dots on the left side of the plot (lower entropy) refer to URLs that have mainly “local” diffusion, while URLs on the right (higher entropy) are spread across many different communities

The *U* and BotScore scores do not seem to be strongly related, even though they show their own peculiarities in terms of how they are distributed across the different communities. It is interesting, though, to uncover the role these user features play in the diffusion of URLs on the network. We, therefore, consider the set of ≈700 URLs in our dataset that were shared more than 100 times. These URLs spread all across the network; the extent of their diffusion is quantified not only by the number of retweets but also by an entropy measure, described Sect. 2.3, that indicates the heterogeneity of the reach of the URLs in terms of the number of different communities that retweet it. Thus, we are able to characterise each URL on three different dimensions: entropy *H*, number of retweets, and features (BotScore, Untrustworthiness) of the users sharing it. In Fig. 3 we cross-check these dimensions to evaluate the interplay between the *U* and BotScore scores and their impact on URL diffusion. For each URL we compute the average *U* and BotScore scores for all users that shared it. There is a clear shift in the Untrustworthiness as the BotScore rises. A cloud of red dots, all located above $\mbox{BotScore} \gtrsim 0.25$, tells us that, on average, the URLs retweeted by users with higher BotScore are often retweeted by users with high Untrustworthiness, which suggests an interesting correlation between these two dimensions. Entropy also comes into play: The highest number of darker red dots (high-*U* URLs) are found in the low-entropy area of the plot. This means that URLs shared by untrustworthy users are likely to gain visibility in a single cluster, or very few communities, instead of being diffused on a larger (global) scale.

**Figure 4:**
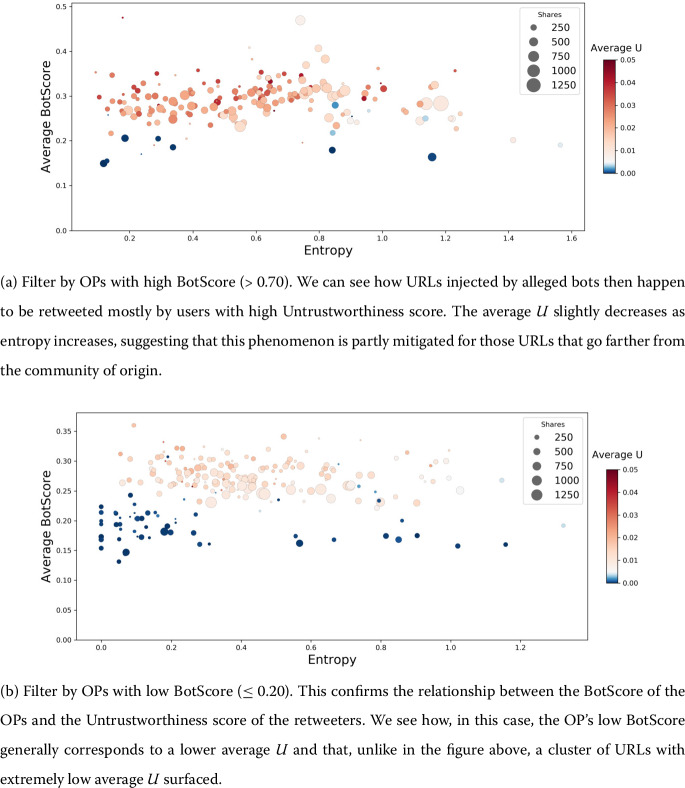
Relationship between URL entropy, Untrustworthiness and BotScore of retweeting users for URLs originally shared by OPs with high BotScore (>0.70 - above), and low BotScore (≤0.20 - below)

We investigate the dynamics of diffusion by further exploring the interplay between *U* and BotScore with a focus on the role of the OPs. Similar to Fig. 3, in Fig. 4a we consider the URL entropy, the average BotScore, and the average Untrustworthiness of retweeting accounts, but this time for all URLs whose OPs have a very high BotScore ($BS>0.70$). The URLs injected into the network by the alleged bots seem to point to very low credibility outlets, or at least are shared mostly by high *U* users. For higher entropy values, the average Untrustworthiness decreases, which suggests that the phenomenon is mitigated for the URLs that go farther away from the community of origin. A counter-check can be obtained if we perform the same analysis, symmetrically, on all URLs injected by users with low BotScore (≤0.2), as shown in Fig. 4b.

**Figure 5:**
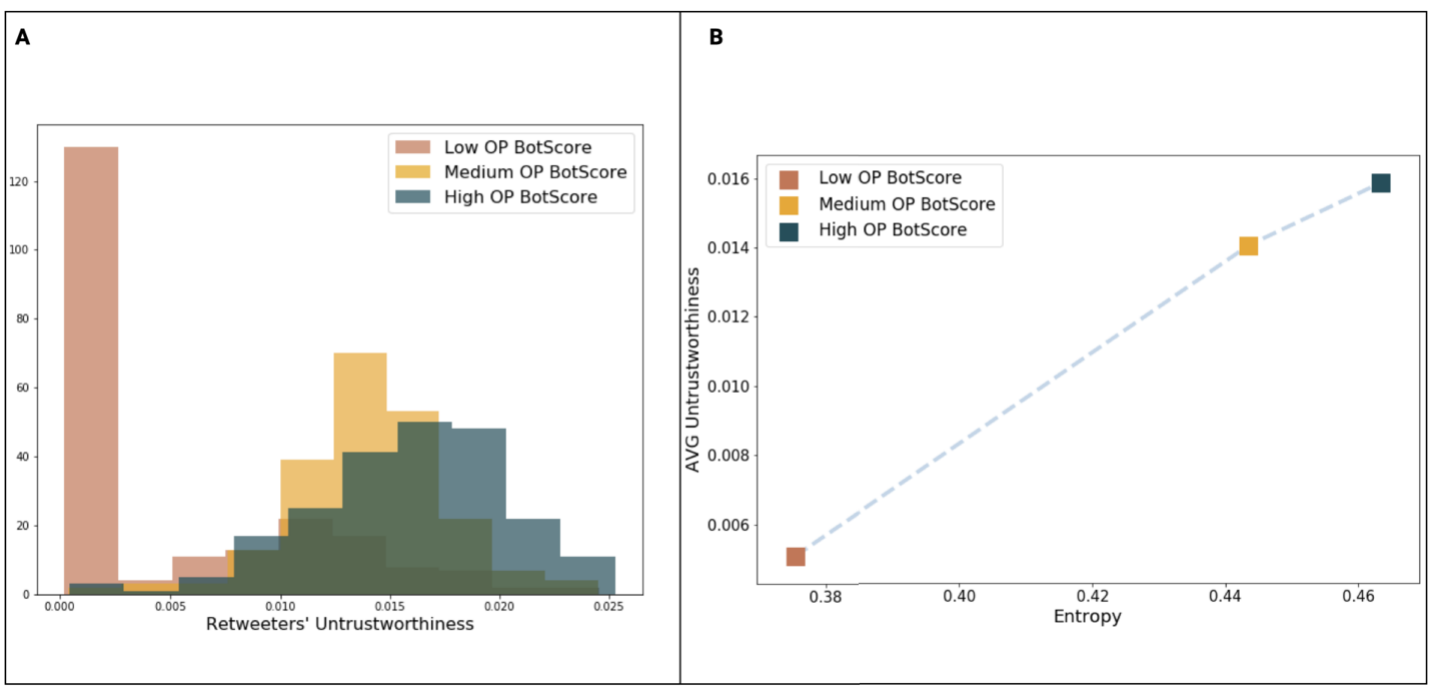
(**A**) Distribution of the Untrustworthiness of the retweeters disaggregated by the BotScore of the OPs. BotScore is divided into tertiles. A shift to the right can be observed as we go up the quantile ladder, displaying once again a positive relationship between the two measures; (**B**) Relationship between Entropy, average Untrustworthiness, and BotScore of the OPs. BotScore is divided into tertiles; once again, a positive relationship between the measures involved can be observed

Figure 5 further corroborates these observations by showing how the distribution of the retweeters’ *U* shifts to the right as the OP’s BotScore increases (Fig. 5(a)); the content injected into the network by alleged bots is not only retweeted by high-*U* users, but it also diffuses across many different communities (Fig. 5(b)).

The combination of these analyses indicates a strong, positive relationship between the BotScore of the OP and the Untrustworthiness of the user that subsequently retweets the URL. In light of this, we argue that users with higher Untrustworthiness are, in general, keener than others to retweet posts first shared by suspiciously bot-like accounts.

### Probability of success

A URL is defined as *successful* if it falls in the fourth quartile (among the top 25% of the most retweeted URLs) of the distribution of the number of retweets per URL in our dataset. We analyse the impact of the OPs on the diffusion of content by quantifying the probability of success of a URL given the BotScore and Untrustworthiness of its OPs.

Note that one URL can have more than one OP by counting all users who tweeted it and are the seeds of different, independent retweet cascades. In such cases we consider the average BotScore and *U* of all the OPs for each URL.

Based on this, we use Bayes theorem to compute the conditional probability that a URL is successful if the average BotScore of its OPs is above a certain value *x* as follows:

3 $$ P(RT \geq t \mid \mbox{avg. BotScore}\geq x) = \frac{P(\mbox{avg. BotScore}\geq x \mid RT \geq t ) \cdot P(RT \geq t)}{P(\mbox{avg. BotScore}\geq x)}, $$

where *RT* is the number of retweets of a URL and *t* is the fixed-threshold number of retweets corresponding to the fourth quartile. The same applies to the success probability conditioned upon the average OP Untrustworthiness:

4 $$ P(RT \geq t \mid \mbox{avg. } U \geq x) = \frac{P(\mbox{avg. } U \geq x \mid RT \geq t ) \cdot P(RT \geq t)}{P(\mbox{avg. }U \geq x)}. $$

**Figure 6:**
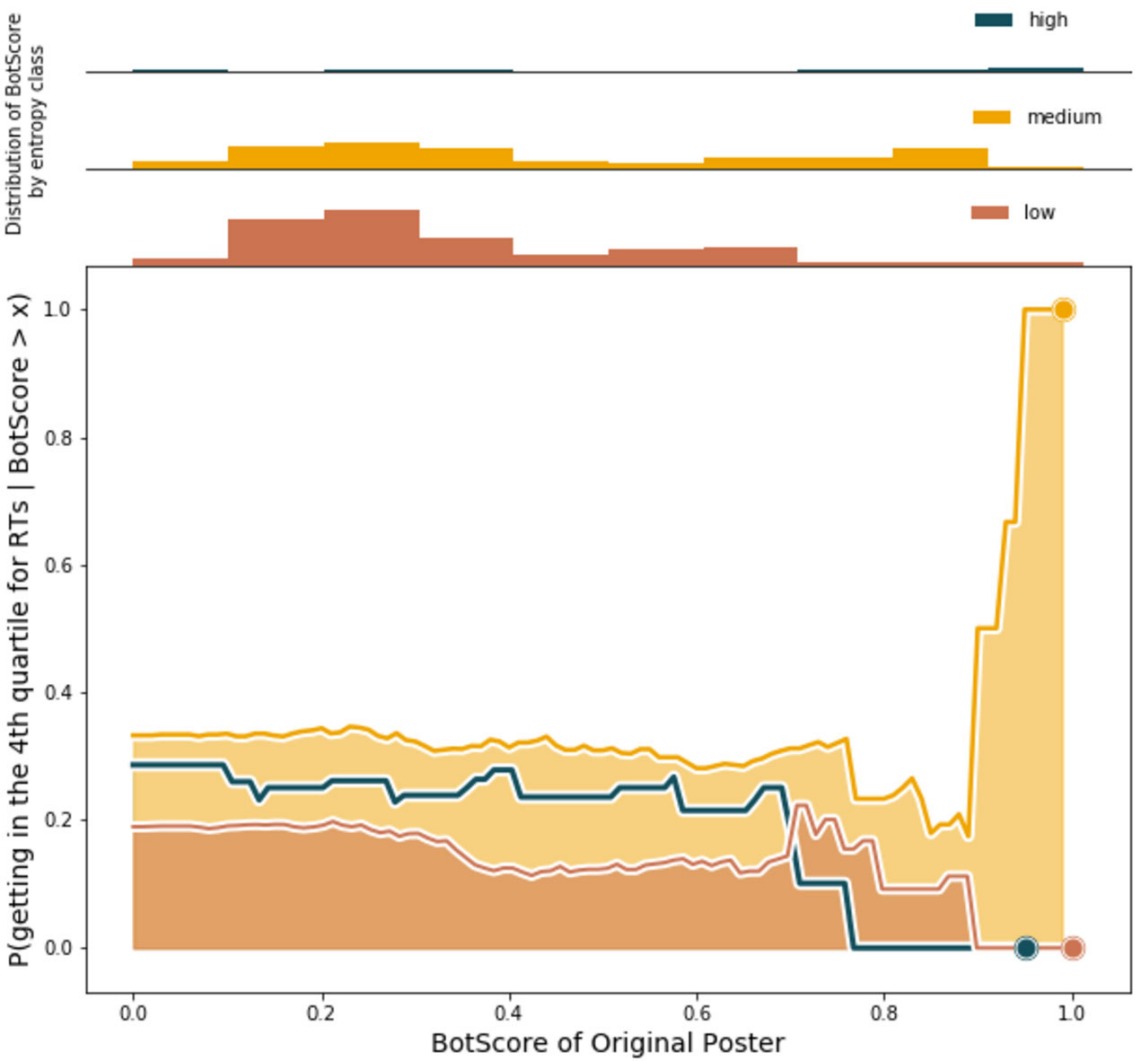
Probability of success for a URL given the BotScore of the OP. Medium entropy URLs tend, in general, to be more successful than the others, regardless of the BotScore of the OPs

**Figure 7:**
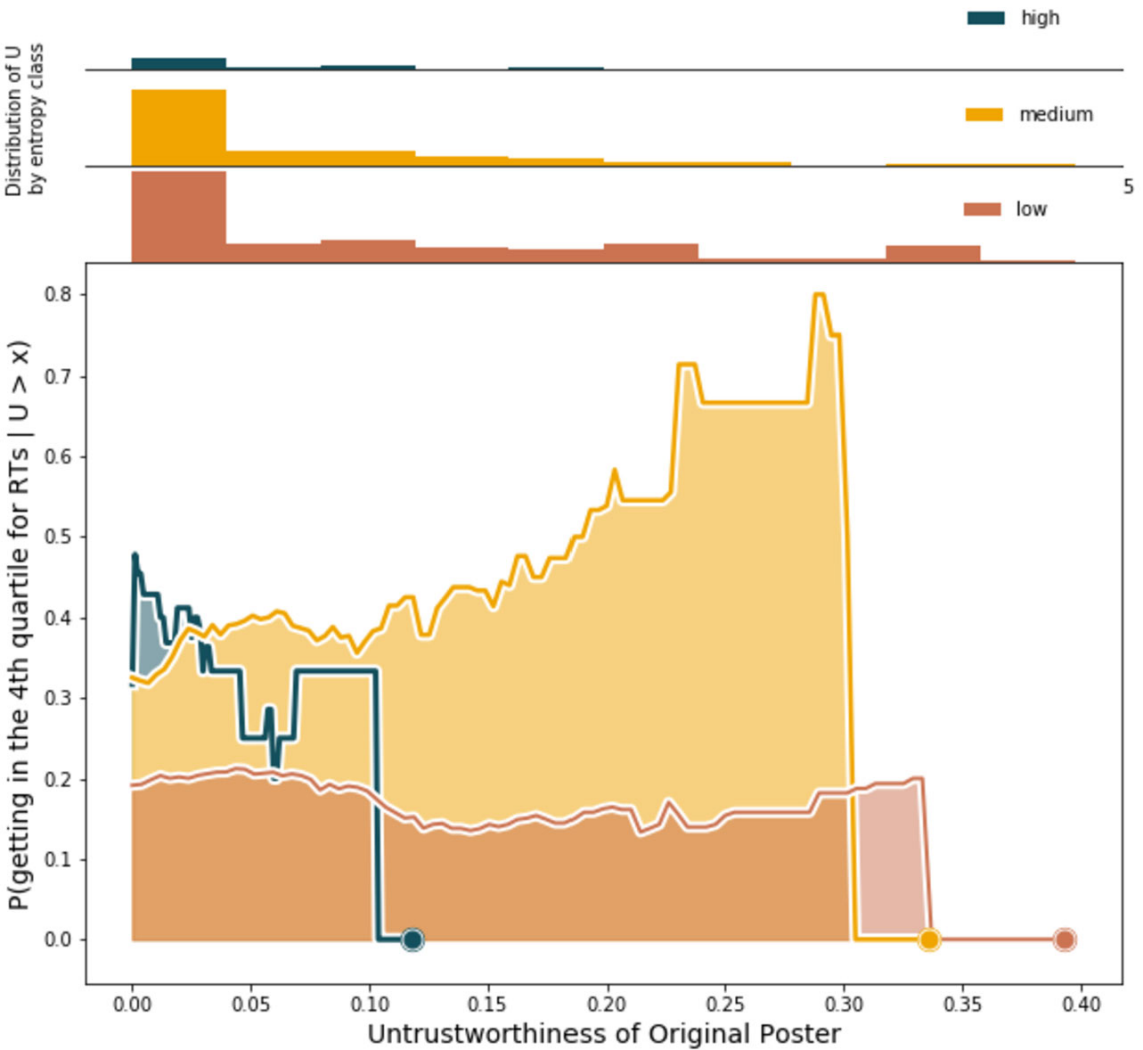
Probability of success of a tweet given the Untrustworthiness of the OP. As seen in Fig. 6 for the BotScore, medium entropy URLs tend to dominate over the others, almost always obtaining more success. The only exception being the high entropy and low-*U* URLs that probably represent a core of reliable, mainstream media content that are widely diffused through the network. Evidence in this Fig. and in Fig. 6 suggests that the key for a URL to be successful is to be diffused beyond its originating community, but without getting too far from it, or better yet, without being shared in too many other communities

In Figs. 6 and 7 we see how the probabilities, defined respectively in Eqs. (3) and (4), vary as functions of the threshold variable *x*, with fixed *t*. Each plot displays the probabilities for the URLs with low (≤0.4), medium (≤0.9), and high (>0.9) entropies. The upper part of each figure shows the distribution of the features (BotScore and *U*) by entropy class, which can unveil interesting patterns. Different probabilities of success for each entropy class implies that broader diffusion of a piece of content, among many different clusters, could have either positive or negative influence on how many times that content is retweeted. High retweet volumes for low-entropy URLs hint that some sort of echo-chamber effect is going on in certain clusters; on the contrary, high retweet volumes for high-entropy URLs imply that crossing the borders of a single community increases the possibility for the content to be successful.

By checking the distributions of BotScore and *U* by entropy class, we see that the high entropy category is, in general, scarcely populated. This has practical implications for the high-entropy probability because there are fewer data points we can use to calculate it. This is particularly evident in Fig. 7, where the complete absence of URLs with high entropy and average OP Untrustworthiness ≳0.10 abruptly brings the probability to 0.

The entropy class clearly seems to discriminate different levels of success. In both cases, medium entropy URLs are those getting the most retweets, gaining more success, showing that *medium* entropy—the diffusion of a URL across different communities, but *not too many*—is a key factor in success. Entropy overshadows the effect of *U* and BotScore over the eventual success of a URL because for each entropy class, the probabilities remain essentially constant. This suggests that BotScore and *U* do not play a key role in defining the “faith” of the diffusion of the URL; there are likely other factors affecting such dynamics, and entropy definitely seems to be one of them.

## Discussion

The methodology described in this paper provides a flexible and robust framework to address the research questions enumerated in the introduction. These research questions can be addressed in real world settings as highlighted by the TWITIMM dataset example with data collected on a controversial topic and the retweet network generated through these data.

Starting the example at research question R1, by calculating the Untrustworthiness index *U* on the community structure of the retweet network, we notice that there are peculiar trends for the different clusters, suggesting that some groups show a more significant circulation of low-credibility media outlets than others. This is particularly true for community *RT2*, previously identified as a community with negative stance towards immigration [3], which is centred around the accounts of unreliable media outlets.

By applying the Botometer [33] to assess the distribution of bots among different clusters, we are able to answer the second research question, R2. Even though we do not find a particularly high presence of bot-like activity in TWITIMM compared to the neutral baseline TWITA, in the cluster analysis we find three communities, *RT2, RT3,* and *RT5*, that display slightly higher right tails, showing a higher presence of bot-like accounts in these communities. This specific analysis relies on the good performance of the language-independent model used for classification; most importantly, it does not necessarily imply a malicious nature in the automated accounts.

It is particularly interesting to study the interplay between *U* and BotScore in content diffusion in addressing R3. It also helps us to compute entropy measures for every URL shared on the network that show whether they are shared by many different communities or remain confined within few clusters, as described in Sect. 2.3. By comparing these measures for all of the users involved in posting and then retweeting media content coming from either low or high credibility outlets, we see in Fig. 3 that the BotScore of the users acts as a good discriminating feature. We notice that for $\mbox{BotScore}\gtrsim 0.25$ the URLs are shared by users with high *U*, especially for the low entropy URLs that are not retweeted by many different clusters. These URLs seem to be where two aspects, bot-like activity and engagement with low-quality information, strongly come together.

Furthermore, we analyse the particularly interesting role of the OPs. It reveals the positive relationship between OP BotScore and the average Untrustworthiness of the retweet events that follow their posts: Users with higher *U* are, in general, keener than the others to retweet posts that are first shared by bot-like accounts. This tendency is even more evident in Fig. 5(a), where we clearly see a shift to the right of the distribution of *U* as the BotScore of the OP increases. Simply speaking, even if we have evidence that accounts with high BotScores have a role in injecting low-credibility content into the network, humans are (still) to blame for generating the success of low-quality information. This low-quality information, as we gather from Fig. 5(b), is also diffused across many different communities.

Finally, to respond to R4, we check how the probability of success of a piece of media content changes as a function of *U*, BotScore, and entropy. Having defined *success* simply as the condition of being among the top 25% of most retweeted URLs, we compute the conditional probability for a URL to fall in this region given the *U* and BotScore of its OPs. Unexpectedly, we see that neither BotScore nor *U* seem decisive in determining the success of a URL: The probabilities follow very similar trends (Figs. 6 and 7). Entropy stands out instead; for both probabilities the class of medium entropy URLs emerges clearly, keeping a high probability throughout the whole range of thresholds set for BotScore and *U*, and completely dominating in the high values. The relevant result, according to our data, is that the key for a URL to be successful is for it to be diffused beyond its originating community but without getting too far from it or, better, without being shared in too many other communities.

The present work has some limitations to acknowledge and some talking points to address. Particularly:

- The conclusions of the empirical case study are, by definition, strongly dependent on the data and therefore not straightforward to generalise. Indeed, Twitter Stream APIs come with some constraints that could, in principle, limit the representativity of the dataset. Even so, we believe that the chosen dataset is a good representation of the debate around immigration in Italy, as it has also been discussed in [3, 16]. Still, this case study provides a showcase of the simplicity and the flexibility of our method, that it can be easily applied to any kind of network of interactions involving the diffusion of online media content.
- The Untrustworthiness index *U* is based on the selection of reliable and unreliable information outlets. Therefore, special care should be taken when selecting the sources that allow us to label the outlets. This coarse grained classification of what is reliable and unreliable cannot always be transferred to the published content. A reliable outlet can publish some unverified rumours or a piece of misinformation, and an unreliable website can occasionally produce true news. Still, relying on an external annotation can be seen as a strength because the credibility check on news media is performed independently and is validated by both the scientific community and public opinion.
- The detection of alleged automated accounts has been performed using the Botometer. It, as any other automatic detection tool for social accounts, presents a number of intrinsic limitations that has been criticised by some authors [35, 36]. However, far from being a tool that perfectly discriminates bots from humans, it asks for extra-precautions to operate it in the best possible way [36] and to exploit the full potentialities of such tools, because results of the classification could be flawed or inaccurate. For this reason it is always important to perform a manual validation of the results (see Sect. A.2), and checks on the temporal consistency of the Botometer’s annotations.
- On the same note, we would like to reemphasise that the disinformation phenomenon is extremely complex and multi-faceted. We have referred to third-party watch-lists to distinguish between high and low credibility media, that is, reliable and unreliable sources of information. Nonetheless, this is a simplification that does not account for other aspects of disinformation. Malicious content can also be generated or amplified by well-known and trusted news media, with varying intentionality. Ambiguous behaviours such as click-baiting, inaccurate titles, and sensationalist tones are not a prerogative of unreliable or alternative media outlets only. This scenario is a very different from what we have pursued here and likely requires a different approach, one focused on the individual news stories rather than on the media outlet. Here we decided to cover the other end of the wide spectrum of disinformation phenomena, to focus on unveiling different patterns of news consumption between reliable and unreliable media outlets, as we have extensively argued in Sect. 1.

## Conclusions and future work

In the age of social media, studying information consumption patterns is crucial to quantify the effective prevalence of low-quality information. To this end, we defined the *Untrustworthiness* index, a simple measure to quantify the engagement of social media users with unreliable media content, that is, the digital-born media that have been identified as consistent disinformation spreaders by external fact checkers. We conducted an empirical analysis to test this method on real world data, evaluating the presence and popularity of low credibility media content in the Italian Twitter debate on immigration. This helped us fulfill the research questions outlined in Sect. 1.2, as we found the index to be a good characterisation of clusters of users of an online social network. Interestingly, when analysing the diffusion of content on the network and the heterogeneity of reach of a piece of news, it emerges that users with a higher *U* appear to be more keen to share media content that was originally published by accounts displaying bot-like automation to some extent. In a way, these findings are in line with other very recent work arguing that “partisan audience diversity is a valuable signal of higher journalistic standards” [32]. Indeed, we find a strong match between community structure, political orientation and circulation of unreliable news, with many users with high *U* localised in few, politically like-minded communities. We conclude, as did the above mentioned paper, that it is crucial to properly design news ranking and filtering algorithms to reach a more diverse audience, to exploit media pluralism instead of succumbing to the so called “echo chambers.”

Furthermore, a high-level study of the global dynamics of content success does not tell us that Untrustworthiness and BotScore are decisive in determining virality. Nonetheless, a detailed reconstruction of the actual retweet cascades could definitely be helpful in developing a more precise idea of the role of bots in the spread of disinformation, expanding the insights gathered in our experiment.

Another aspect that could be explored is how different classes of networks can be studied, according to distinct user interactions. In line with other work [37, 38] that considers Twitter as a multi-layer network, understanding the preferred interaction mechanism (e.g., retweets, mentions) to share information from high or low credibility URLs could shed new light on news consumption patterns on social media. This would further support the understanding of misinformation and disinformation diffusion.

## Appendix:  Supplementary material

#### A.1 Distribution of high and low credibility news media outlets in the TWITIMM dataset

One of the main methodological contributions of this work is the Untrustworthiness Score, a quantity that measures the level of engagement with reliable and unreliable news media outlets of each user. As explained in Sect. 3.2, for the specific case study of the Italian Twitter debate on immigration, these outlets are selected by referring to third-party sources. Low credibility media outlets are obtained from two well-known debunking sites, forming list *L*⊖; high credibility media are selected from the Audiweb 2019 reports,^6^ without blacklisted sites (*L*⊖) forming list *L*⊕. As already specified, this ensures that all the websites in *L*⊕ have not been flagged as consistent spreaders of malicious content.

In this appendix we provide more information about how URLs from *L*⊕ and *L*⊖ are distributed in our dataset. Particularly, in Fig. 8 we see the popularity of each URL as a function of its ranking.

Figure 8Frequency of the URLs as a function of the ranking, where $\mbox{ranking}=1$ is assigned to the most retweeted URL in the dataset. The distribution is highly heavy-tailed with 186 out of 700 URLs to the left of the red line accounting for 50% of the total URL shares. The coloured bars represent the distribution of reliable, unreliable, and other (i.e., unlisted) URLs. Only reliable and unreliable URLs contribute to the computation of the Untrustworthiness index

The distribution is heavy-tailed; of around 700 URLs, 186 account for 50% of the total number of shares. On both sides of the red line, the distribution of URLs from reliable (*L*⊕), unreliable (*L*⊖), and other (i.e., unlisted) media sources are roughly the same, with a very slight prevalence of unlisted and unreliable sources over the high credibility ones. Only *L*⊕ and *L*⊖ contribute to the computation of the Untrustworthiness index because we are not able to tell anything about the unlisted media outlets. The outlet lists *L*⊕ and *L*⊖ are reported in Table 4 and in Table 3, respectively.

**Table 3:** List of unreliable media outlets, i.e., online newspaper that have been reportedly considered to contribute spreading mis- and dis-information, according to “*butac.it*” and “*bufale.net*”

*L*⊖ Web Domains
www.breaknotizie.com
www.byoblu.com
comedonchisciotte.org
www.il-giornale.info
www.ilpopulista.it
www.il-quotidiano.info
www.ilprimatonazionale.it
www.imolaoggi.it
informarexresistere.fr
italianosveglia.com
www.jedanews.it
www.lonesto.it
www.riscattonazionale.org
www.saper-link-news.com
www.silenziefalsita.it
www.skynew.it
www.stopeuro.news
tg-news24.com
www.tg24-ore.com
tg5stelle.it
www.ticinolive.ch
tuttiicriminidegliimmigrati.com
vocedelweb.com
voxnews.info
zapping2017.myblog.it

**Table 4:** List of the 100 most accessed media outlets according to “www.audiweb.it” in 2019, already filtered by sites in *L*⊖. As an empirical counterpart of “unreliable” outlets, we consider these websites “reliable”

*L*⊕ Web Domain	
repubblica.it	dagospia.com
twitter.it	la7.it
corriere.it	nostrofiglio.it
virgilio.it	notizie.it
gazzetta.it	deejay.it
upday.com	it.businessinsider.com
tgcom24.mediaset.it	formulapassion.it
libero.it	wired.it
ilmessaggero.it	deabyday.tv
ilfattoquotidiano.it	ticketone.it
fanpage.it	caffeinamagazine.it
leggo.it	milanofinanza.it
lastampa.it	elle.com/it/
tuttomercatoweb.com	treccani.it
giallozafferano.it	focus.it
sport.sky.it	corriereadriatico.it
ansa.it	grazia.it
liberoquotidiano.it	ilbianconero.com
ilgiornale.it	lacucinaitaliana.it
calciomercato.com	105.net
huffingtonpost.it	lanuovasardegna.it
my-personaltrainer.it	alvolante.it
bendingspoons.com	lagazzettadelmezzogiorno.it
espresso.repubblica.it	zingarate.com
ilmattino.it	viamichelin.it
italiaonline.it	studenti.it
ilsole24ore.com	rockol.it
donnamoderna.com	lasicilia.it
vanityfair.it	ilcentro.it
corrieredellosport.it	supereva.it
tuttosport.com	blitzquotidiano.it
tpi.it	cosmopolitan.it
tg24.sky.it	gazzettadelsud.it
ilgazzettino.it	lettera43.it
ilpost.it	ilgiornaledivicenza.it
dailymotion.com	larena.it
raiplay.it	wetransfer.com
mediasetplay.mediaset.it	prealpina.it
adnkronos.com	discoveryplus.it
notizie.tiscali.it	filmtv.it
eurosport.it	rai.it
tim.it	quotidianodipuglia.it
it.altervista.org	iltempo.it
rainews.it	ilmiolibro.it
unionesarda.it	marieclaire.com
mymovies.it	glamour.it
affaritaliani.it	vogue.it
greenme.it	termometropolitico.it
gds.it	esquire.com

Finally, the top 15 web domains by shares are reported in Table 5, together with their classification (reliable, unreliable, or other).

**Table 5:** List of the top 15 domains by shares

Domain	Flag	Shares
www.imolaoggi.it	unreliable	15,105
www.liberoquotidiano.it	reliable	10,500
www.repubblica.it	reliable	9079
www.ilgiornale.it	reliable	6872
www.riscattonazionale.org	unreliable	3874
www.ilfattoquotidiano.it	reliable	3386
www.lastampa.it	reliable	3338
voxnews.info	unreliable	3200
www.ilprimatonazionale.it	unreliable	3098
www.ansa.it	reliable	2428
huffp.st	reliable	2393
www.corriere.it	reliable	2090
www.secoloditalia.it	other	1728
www.tgcom24.mediaset.it	reliable	1711
it.blastingnews.com	other	1704

#### A.2 Validation of the Botometer’s botscore

In Sect. 3.3 we introduced the BotScore, a numerical score that represents the probability that an account is automated. This score is computed through the *Botometer* [33], a tool developed by the researchers at OSoMe, the Observatory on Social Media at Indiana University. The BotScore ranges from 0 (scarce) to 1 (extremely high) probability of automation. Before using the *Botometer* to evaluate each user in our dataset, we cross-checked the tool’s prediction accuracy on a small sample of accounts, following the methodology in [36]. We performed a stratified sampling of our dataset, according to the distribution of the BotScore, and manually checked 150 accounts, looking for signs of bot-like activity. The annotation was carried out by three annotators and the final label was chosen based on a majority rule. The results are displayed in Fig. 9. More than half of the alleged bots according to the human annotation (the red dots) are above $\mbox{BotScore} > 0.36$, a symbolic threshold that, in our dataset, accounts for 80% of the users. Almost all the red dots are above $\mbox{BotScore} > 0.2$, the 60% threshold of users. We concluded that the evaluation made by the *Botometer* may disagree with the human annotators’ evaluation on some accounts, but it can still provide valuable information. It is not granted that an account with a BotScore above an arbitrary threshold is actually automated, but in our opinion it is safe to assume that accounts that show bot-like behavioural patterns are mostly among those with high BotScore. This is consistent with the *Botometer* guidelines and with the purpose of our analysis.

Figure 9The Botometer vs human annotator evaluation of a small sample of accounts. More than half of the accounts that have been flagged as possible bots by human annotators (red dots) have a $\mbox{BotScore} > 0.36$. All the accounts that show patterns of automation have a $\mbox{BotScore} > 0.20$

In Fig. 10 we see the complementary cumulative probability (CCDF), the probability of detecting bot-like activity for BotScore≥ x, computed as the proportion of accounts with BotScore≥ x that were manually annotated as alleged bots. This plot tells us that, based on our manual annotation, it is more likely to detect bot-like activity for higher values of the BotScore.

Figure 10Probability of detecting bot-like activity for $\mbox{BotScore}\geq x$, computed as the proportion of accounts with $\mbox{BotScore}\geq x$ that were manually annotated as alleged bots: the higher the BotScore, the higher the chance of detecting bot-like activity

We also checked for the temporal consistency of the score by comparing our score (December 2020) to the current one (end of September 2021) for the accounts in the stratified sample. We found a strong linear correlation (Pearson’s $\mathrm{coefficient} = 0.84$, $p=2.8\cdot 10^{-38}$).

## Data Availability

Twitter data is currently available upon request to the authors; tweet IDs will be made available in a Github repository, in accordance with Twitter policies on data sharing.

## Footnotes

1 See the Reporters Without Borders’s report “Contribution to the European Union public consultation on media pluralism and democracy”, July 2016, at *shorturl.at/jBCMT*, last access: Oct. 3, 2021

2 See “2021 Trust Barometer Global Results.”, Available at *shorturl.at/vJMP0*, last access: Oct. 3, 2021.

3 The TWITA dataset is an ongoing collection of tweets identified as being written in Italian; the collection comprises hundreds of millions of tweets, starting from February 2012, with no filter but the language.

4 http://www.audiweb.it/

5 In this specific case study, $L^{\oplus}$ is obtained by filtering out websites $\in L^{\ominus}$ from the Audiweb list. This does not ensure that all the websites obtained are entirely reliable: we know that they have not been flagged as consistent spreader of malicious content by the chosen observers.

6 http://www.audiweb.it/

## Abbreviations

RT: Retweet
U: Untrustworthiness Score
OP: Original Poster
