# Supplementary figures and images for: Measuring user engagement with low credibility media sources in a controversial online debate
Source: EPJ Data Sci. 2022 May 16;11(1):29. doi: 10.1140/epjds/s13688-022-00342-w (PMC9108351; doi:10.1140/epjds/s13688-022-00342-w)

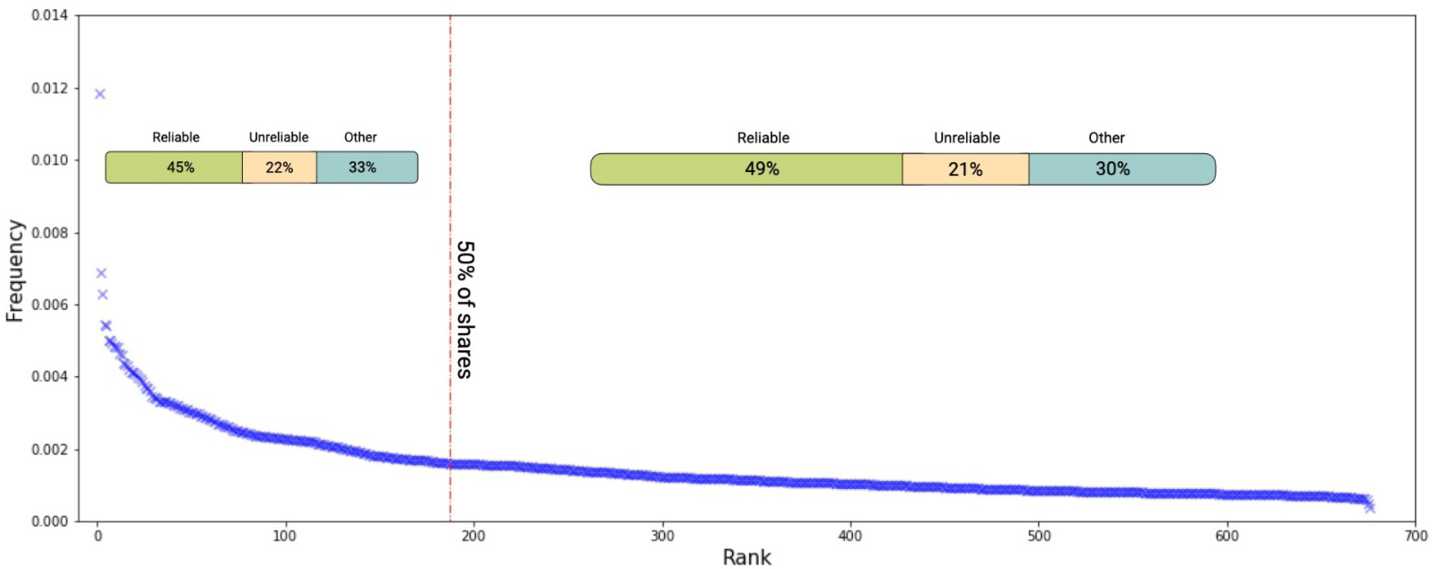

Supplement: Supplementary file 1 — Frequency of the URLs as a function of the ranking, where $\mbox{ranking}=1$ranking=1 is assigned to the most retweeted URL in the dataset. The distribution is highly heavy-tailed with 186 out of 700 URLs to the left of the red line accounting for 50% of the total URL shares. The coloured bars represent the distribution of reliable, unreliable, and other (i.e., unlisted) URLs. Only reliable and unreliable URLs contribute to the computation of the Untrustworthiness index [file 13688_2022_342_Fig8_HTML.jpg]

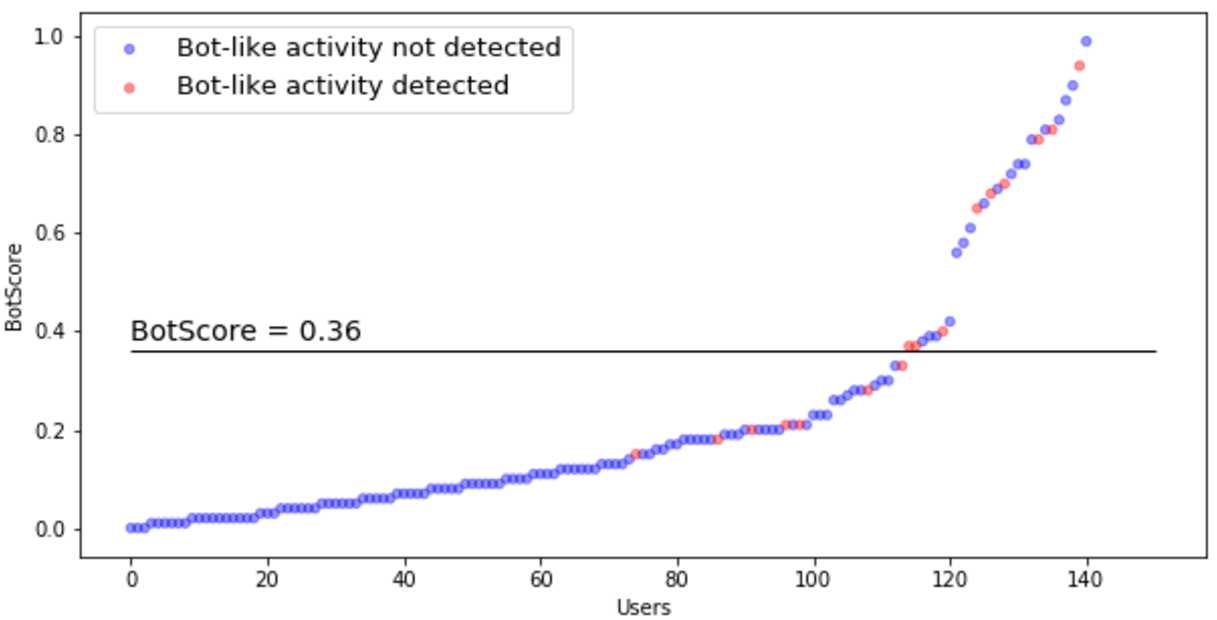

Supplement: Supplementary file 2 — The Botometer vs human annotator evaluation of a small sample of accounts. More than half of the accounts that have been flagged as possible bots by human annotators (red dots) have a $\mbox{BotScore} > 0.36$BotScore>0.36. All the accounts that show patterns of automation have a $\mbox{BotScore} > 0.20$BotScore>0.20 [file 13688_2022_342_Fig9_HTML.jpg]

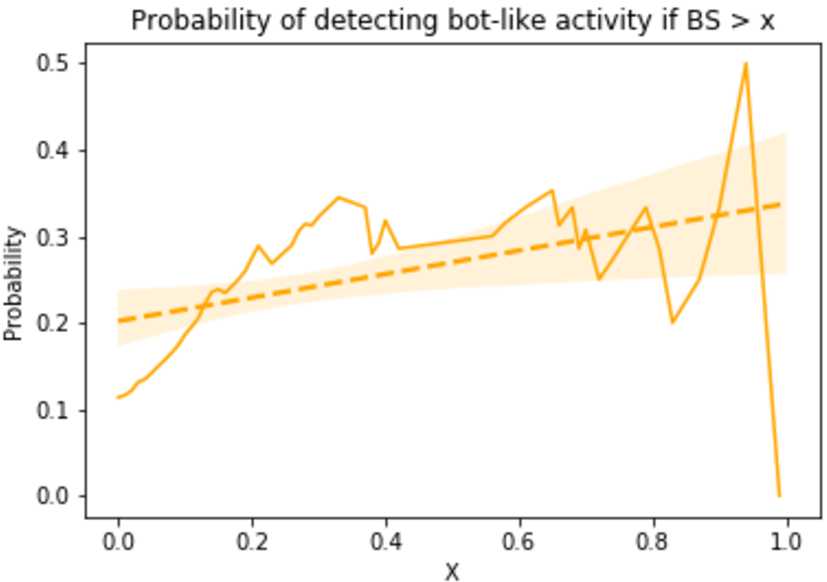

Supplement: Supplementary file 3 — Probability of detecting bot-like activity for $\mbox{BotScore}\geq x$BotScore≥x, computed as the proportion of accounts with $\mbox{BotScore}\geq x$BotScore≥x that were manually annotated as alleged bots: the higher the BotScore, the higher the chance of detecting bot-like activity [file 13688_2022_342_Fig10_HTML.jpg]
